# Episiotomy Practice and Its Associated Factors in Africa: A Systematic Review and Meta-Analysis

**DOI:** 10.3389/fmed.2022.905174

**Published:** 2022-06-24

**Authors:** Beshada Zerfu Woldegeorgis, Mohammed Suleiman Obsa, Lemi Belay Tolu, Efa Ambaw Bogino, Tesfalem Israel Boda, Henok Berhanu Alemu

**Affiliations:** ^1^Department of Internal Medicine, Wolaita Sodo University, Sodo, Ethiopia; ^2^Department of Anesthesia, Arsi University, Asella, Ethiopia; ^3^Department of Obstetrics and Gynecology, Saint Paul's Hospital Millennium Medical College, Addis Ababa, Ethiopia; ^4^Dermatovenereology Department, Wolaita Sodo University, Sodo, Ethiopia

**Keywords:** Africa, associated factors, delivery, episiotomy, parturient, perineum

## Abstract

**Background:**

Episiotomy, a surgical procedure that enlarges the vaginal opening during childbirth, was common practice until the early 2000s. Other sources, including the World Health Organization (WHO), advocate for the selective use of episiotomy. Episiotomy rates, on the other hand, have remained high in developing countries, while declining in developed countries. As a result, the current study sought to determine the overall prevalence of episiotomy in Africa as well as the risk factors associated with its practice.

**Methods:**

Articles were searched in international electronic databases. A standardized Microsoft Excel spreadsheet and STATA software version 14 were used for data extraction and analysis, respectively. The Preferred Reporting Items for Systematic Reviews and Meta-Analysis (PRISMA) checklist was used to write this report. A random-effects meta-analysis model was used to determine the pooled prevalence of episiotomy. A heterogeneity test was conducted using I-Squared (*I*^2^) statistics. Egger's test and funnel plots were conducted to detect publication bias. Subgroup analysis was also conducted. Association was expressed through a pooled odds ratio (OR) with a 95% Confidence Interval (CI).

**Result:**

A total of 21 studies with 40,831 participants were included in the systematic review and meta-analysis. The pooled prevalence of episiotomy practice was 41.7% [95% CI (36.0–47.4), *I*^2^ = 99.3%, *P* < 0.001). Primiparity [OR: 6.796 (95% CI (4.862–9.498)), *P* < 0.001, *I*^2^: 95.1%], medical doctors- assisted delivery [OR: 3.675 (95% CI (2.034–6.640)), *P* < 0.001, *I*^2^: 72.6%], prolonged second stage of labor [OR: 5.539 (95% CI (4.252–7.199)), *P* < 0.001, *I*^2^: 0.0%], using oxytocin [OR: 4.207 (95% CI (3.100–5.709)), *P* < 0.001, *I*^2^: 0.0%], instrument -assisted vaginal delivery [OR: 5.578 (95% CI (4.285–7.260)), *P* < 0.001, *I*^2^: 65.1%], and macrosomia [OR: 5.32 (95% CI (2.738–10.339)), *P* < 0.001, *I*^2^: 95.1%] were factors associated with episiotomy practice.

**Conclusion:**

In this review, the prevalence of episiotomy among African parturients was high. A selective episiotomy practice should be implemented to reduce the high episiotomy rates.

**Systematic Review Registration:**

https://www.crd.york.ac.uk/prospero/display_record.php?ID=CRD42021293382, identifier: CRD42021293382.

## Background

An episiotomy is a vaginal and perineal surgical incision performed by a skilled birth attendant, to widen the vaginal opening (1, 2), late in the second stage when the perineum is stretched thin (3), and it is one of the most commonly performed surgical procedures all over the world (4). There are seven different ways to perform an episiotomy, with the two most common types in the literature and medical practice being “midline” and “mediolateral.” A midline (sometimes called “median”) episiotomy is a vertical incision from the posterior fourchette that runs along the midline through the central tendon of the perineal body. A mediolateral episiotomy is an incision beginning in the midline and directed laterally and downwards away from the rectum (2).

This surgical procedure is not without consequences as compared to permitting the perineum to tear. To begin with, episiotomy might be detrimental with respect to urinary incontinence symptoms (5). In a study conducted to assess the impact of episiotomy on the urogenital hiatus using transperineal ultrasound, the urogenital hiatal area was altered by episiotomy (6). In contrary, indicated use of episiotomy resulted in a significant decrease in third and fourth degree lacerations in a population-based observational study in Texas, United States of America (7).

According to a Cochrane database systematic review, women who had selective episiotomy experienced 30% less severe perineal trauma at birth than women who had a routine episiotomy policy. In terms of Apgar scores of <7 at 5 min, the number of women developing perineal infection, the number of women reporting painful sexual intercourse 6 months or more after delivery, and urinary incontinence 6 months or more after delivery, there was probably no or little difference reported. However, other significant long-term effects and outcomes were not reported in these trials (urinary fistula, rectal fistula, and fecal incontinence). As a result, the rationale for performing routine episiotomies to prevent severe perineal trauma was found to be unjustified, and there were no benefits to the baby or the mother from routine episiotomy (8).

Even when episiotomy technique is considered, mediolateral episiotomy does not appear to be protective against clinically or sonographically diagnosed obstetrical anal sphincter injuries (OASIS), and it was associated with decreased sexual functioning as well as sexual desire, arousal, and orgasm within the first 5 years after delivery (9). Furthermore, in a study conducted in 2015–2016 with the goal of describing the detailed epidemiology of labor and delivery in China, mediolateral episiotomy without indications more than doubled the risk of third and fourth degree perineal laceration in nulliparae without neonatal benefits, remembering the consequences of injudicious use of episiotomy (10). Prophylactic use of episiotomy in critical conditions such as shoulder dystocia, instrumental deliveries, occiput-posterior position, fetal macrosomia and non-reassuring fetal heart patterns don't prevent 3rd or 4th degree perineal tear (11). Nonetheless, a comparative, retrospective, mono-centric study in a university maternity unit in Besançon, France, found that selective episiotomy could reduce the incidence of perineal tears, particularly second-degree perineal tears, without increasing the rate of OASIS (12).

In order to combat the pain correlated with episiotomy, water birth has gained popularity globally, especially in midwifery- led care settings (13). Women's experiences with water birth matched groups in a prospective study by Lathrop et al. revealed that water birth was associated with a decreased likelihood of perineal lacerations (14). Furthermore, water immersion may reduce episiotomy rates (15, 16). Nonetheless, a lack of high-quality evidence clouded informed decisions about the advantages and disadvantages of water birth (17). Therefore, the merit and risks of water birth should be discussed thoroughly with the parturient during the process of informed decision making with mothers interested in this option (16).

Every year, ~140 million babies are born worldwide (18). In 2019, the United Nations (UN) estimated that the total fertility rate of Sub-Saharan Africa (SSA) would be at 4.7 births per woman from 2015 to 2020, which is more than double the level of any other region in the world (19). In concert with this, the rates of episiotomy practice have remained high worldwide, particularly in less industrialized countries and East Asia (20–22).

Reported rates of episiotomies vary greatly from one country to another across the globe. The lowest rate (1%) of episiotomy was reported in Sweden, whereas the highest (100%) was reported in Taiwan among primiparous parturients (23). Several countries have registered higher proportion of episiotomy practice.For instance, 58% in Italy in 1999; 66% in Oman in 2015; 67.5% in Poland in 2010; 68% in India in 2008; 75% in Cyprus;94 % in Cambodia; approximately 95% in Mexico among primiparous women (20, 23–28). In addition, significant number of women undergone episiotomy in Asian countries, 42–98% (23, 29, 30).

Furthermore, despite the standard recommendations that corroborate judicious use of episiotomy, increasing and variable patterns have been reported in Mexico: 41.8% in the state of Oaxaca and 77.2% in Mexico City (8, 25, 31). A sharp decline in episiotomy rates was reported in some countries like Turkey (93.3% in primipara women and 30.2% in multipara women in 2013) (1), France (18.6% from 2013 to 2017) (2), China (85.50% in 2003 to 41.7% in nulliparae and 21.5% in multiparae from 2015 to 2016) (3), the United States of America (20.3% in 2002 to 9.4% 2011 (4). Moreover, in Brazil episiotomy rate around the country declined from ~94% in 2000 (32) to 54% in 2014 (33) and 42% in 2019 (34), in Finland decreased from 71.5% in 1997–1999 to 54.9% in 2006–2007 among primiparous women, and from 21.5% in 1997–2001 to 9.2% in 2006–2007 among multiparous women (35). To summarize, the larger disparity in episiotomy rates around the world, as made evident by historical trends, is closely attributable to differences in episiotomy policies and resources (8, 20).

Individual and clinical factors related to mothers; individual and clinical factors related to the newborn; as well as the socio-demographic profiles of the parturient in Africa and other countries influence episiotomy practice (36). In studies conducted in Brazil (37, 38), Nigeria (39–42),Turkey (43), the United States (44), and Ethiopia (45, 46), the odds of episiotomy practice were positively correlated with younger age at delivery. Nonetheless, advanced maternal age (≥35) was reported as an attributable factor in some studies (37, 47–49).

According to Macleod et al. (50), Koskas et al. (51), Giannella et al. (52), Cromi et al. (48), Beyene et al. (53), Tobiaw Tefera et al. (54), Teshome et al. (46), and Pebolo et al. (55) episiotomy was significantly associated with a prolonged second stage of labor. Furthermore, macrosomia (42, 51, 53, 54, 56–58), advanced gestational age (49, 59, 60), breech presentation (50, 51, 57, 60–62), primiparity (40–42, 45, 46, 53, 56–58, 61, 63–65), oxytocin use (45, 53, 59, 62, 66, 67), meconium-stained amniotic fluid (49, 50), reduced apgar score (67), assisted breech vaginal delivery or vaginal operative delivery (forceps) (41, 42, 53, 54, 56, 58, 61, 63, 66), analgesia (49, 67, 68), non-reassuring fetal heart rate pattern (68), persistent occipito posterior position (41), post-term pregnancy (45), fetal distress (47, 69), perineal tear (66), private character of the mother (41), a history of gestational hypertension (45), birth spacing <2 years (66), vaginal birth after cesarean section (41), maternal under nutrition (64), history of episiotomy in their index delivery (70), and delivery attended by obstetricians and gynecologists (40, 57, 61) were found to be the risk factors documented in these studies. That said, the odds of episiotomy practice may vary within the African context and around the globe, and hence midwifes and obstetricians must better weigh the risks and benefits in order to predict and curb the impacts associated with liberal use of episiotomy (8).

Banta and his associate found four advantages to episiotomy. To begin with, it is claimed that a clean, straight incision is easier to repair and heals faster than a laceration or tear. Second, it is claimed that episiotomy results in fewer third-degree lacerations. Third, episiotomy is said to prevent fetal brain injury by lowering the fetal head's pressure on the pelvic floor. Fourth, episiotomy is said to shorten the second stage of labor, which helps to avoid pelvic floor damage (71). Additionally, episiotomy is justified in preeclampsia (72), in the event of abnormal cardiotocography, inability to control maternal blood pressure, imminent eclampsia, worsening biochemistry, or worsening maternal symptoms, for expeditious delivery of the newborn by shortening the second stage of labor and avoiding suffering for the baby (73). Finally, episiotomy requires laboring mothers to provide informed consent (74). Performing episiotomy without informed consent or with coerced consent is deemed to be instances of obstetric violence (16).

In Africa, although there has been no representative data, the reported rate of episiotomy ranged from 9.3% in a study conducted in South East Nigeria (40) to 73% in Uganda (55). Understanding the magnitude and risks associated with episiotomy can help adhere to existing or develop new protocols that are consistent with World Health Organization (WHO) (75) and American College of Obstetricians and Gynecologists (ACOG) recommendations that emphasize the judicious use of episiotomy (3). To date, there has been no systematic review and meta-analysis conducted to estimate the pooled prevalence and identify risk factors associated with episiotomy practice in Africa. Therefore, the current study aimed to address these two questions: (i) what is the continent's overall estimate of episiotomy practices? (ii) What are the factors that may influence episiotomy practices among African women who give birth in health facilities?

## Methods

### Reporting and Study Protocol Registration

The goal of this systematic review and meta-analysis was to determine the pooled prevalence of episiotomy practice and the factors associated with it among African parturients who gave birth in public health facilities. The study protocol for this study was prepared and registered in the International Prospective Register of Systematic Reviews (PROSPERO) databases on 25/12/2021 (available from: https://www.crd.york.ac.uk/prospero/display_record.php?ID=CRD42021293382) we confirmed the absence of ongoing systematic reviews on this topic by following the guidance note for registering a Systematic Review Protocol to avoid duplication. The meta-analysis was reported using the Preferred Reporting Items for Systematic Reviews and Meta-analysis (PRISMA)-Statement (76) (Supplementary File 1).

### Inclusion Criteria

The inclusion criteria for this review were determined using the CoCoPop mnemonic (condition, context, and population). **Population/Participants** -parturient mothers who were reported to have undergone episiotomy at health facilities in Africa. **Context-**Observational Studies (descriptive and analytic cross-sectional studies, cohort studies, and case control studies) published in English between January 1, 2000 and December 31, 2021, spanning more than two decades due to a scarcity of primary studies. **Condition**-Studies that reported the outcome of interest based on the prevalence and risk factors associated with episiotomy practice were included in this review.

### Exclusion Criteria

We excluded studies without full text access; articles that contained insufficient information; findings from personal opinions; articles reported outside the scope of the outcome of interest; qualitative study design; case reports; case series; letters; and previous systematic review.

### Operational Definitions

#### Episiotomy

It is an obstetric surgical procedure in which incisions are made in the vulva and perineum to allow for a smooth delivery of the newborn by creating enough space (3).

#### Delayed or Prolonged Second Stage of Labor

If the labor lasts longer than 2 h without epidural analgesia or 3 h with epidural analgesia in nulliparous women, or 1 h without or 2 h with epidural analgesia in multiparous women (77).

#### Macrosomia

A new born birth weight ≥4,000 g (78).

#### Oxytocin

Is a drug prescribed for laboring mothers for induction or augmentation of labor by enhancing uterine contraction (78).

#### Parity

Parity is determined by the number of pregnancies reaching the age of viability. A woman who has been delivered only once of a fetus or fetuses born alive or dead with an estimated length of gestation of above the age of viability is termed primiparity. Whereas, multipara is a woman who has completed two or more pregnancies to the age of viability (78).

#### Spontaneous Vertex Delivery

When the fetal presenting part is the vertex or occiput in a laboring mother, labor begins spontaneously and the delivery is accomplished with minimal assistance (78).

### Search Strategy

Our search was restricted to articles published in English from January 1, 2000 to December 31, 2021. The electronic databases of PubMed, Hinari, Science Direct, Web of Science, African Journal of Online (AJOL), Cumulative Index to Nursing and Allied Health Literature (CINAHL), Excerpta Medica database (EMBASE), Google, Journal Storage (JSTOR), and Google scholar were searched. Using the snowballing method, the reference lists of the identified studies were also scrutinized to identify other relevant articles that were not captured during the initial search. We used key concepts to build a search strategy while conducting a comprehensive PubMed search. Initially, Medical Subject Headings (MeSH) terms relevant to our search were identified and added to the search builder. Next, we identified every possible keyword for each key concept and thoroughly used a combination of MeSH and keywords, truncating (^*^) of stems that are four letters or longer, putting double quotes ("“) around any multi-phrase, and adding field tags [tiab] and [tw] for each concept. Finally, after double checking that syntax was correct and Boolean operators were in all caps, we started running a search in the PubMed search box using a building block approach, which means we built the search one concept at a time and then combined concepts together at the end [“women^*^” (text word) OR “pregnant mother” (text word) OR “birth” (text word) OR (“pregnant women” (MeSH Terms) AND “women” (MeSH Terms)] AND [“episiotomy” (text word) OR “episiotomy practice” (text word) OR (“obstetric surgical procedures” MeSH Terms)] AND “episiotomy.” From January 1st to February 30th, 2022, two authors (BW and EA) participated in a double blinded search. The full search results were included as an additional file (Supplementary File 2).

### The Study Selection Procedure

The retrieved studies were exported to EndNote X7, which was then used to remove duplicate studies. After removal of duplicates, two authors (BW and MO) independently screened the titles and abstracts to determine the eligibility of studies. To describe the extent to which assessments by multiple authors are similar, the Cochrane handbook for systematic reviews of interventions was consulted. Values of kappa 0.75 (75 percent) were considered in this way, indicating excellent agreement. The screened articles were then subjected to a full article review by two independent authors (TI and HB). The inclusion and exclusion criteria were used to screen the articles.

### Methodological Quality Assessment

The Joana Briggs Institute (JBI) critical appraisal checklists (79) were used to assess the quality of the studies. The methodological quality of each study was independently evaluated by two reviewers (EA and LT). Discrepancies were solved through discussion with a third independent reviewer (MS.O.). Hence, studies scoring 7 or above after evaluation against these criteria were included in this systematic review and meta-analysis. In this manner, for studies reporting only prevalence data, the following major components were evaluated: appropriateness of the sample frame for addressing the target population, sample size adequacy, study setting and participants, whether the data analysis was conducted with sufficient coverage of the identified sample, validity and reliability of the measurement, appropriateness of the statistical analysis, and adequacy and management of response rate (Supplementary File 3). For the analytical cross-sectional studies, the JBI checklist assessed the following main components: inclusion criteria, participants and settings, whether the exposure was measured in a valid and reliable way, whether the standard and objective criteria were used for measuring the outcome, confounding factors and strategies used to deal with them, whether the outcome was measured in a valid and reliable manner, and appropriateness of the statistical analysis (Supplementary File 4).

### Data Extraction

Using a standard Microsoft Excel spreadsheet, BW and MO independently extracted the relevant data. For data extraction, the JBI adopted formats were used (80). The author's name, study period and year of publication, methods and settings, age of the mothers, sample size and sampling procedure, data collection instrument, estimate of episiotomy practice with 95 percent confidence interval, response rate, and factors associated with episiotomy were all extracted. After retrieving data from 30% of the studies, the reliability agreement among the data extractors was assessed and confirmed using Cohan's kappa coefficient. As a result, the kappa coefficient's strength of agreement was classified as poor (≤ 0.20), fair (0.21–0.40), moderate (0.41–0.60), good (0.61–0.80), and almost perfect agreement (0.81–1) (81) and a kappa statistic value ≥0.5 was considered congruent and accepted. In the case of disagreements between the two data extractors, LT was involved in resolving them through discussion and re-checking of the original articles.

### Summary Measures

The number of parturients who received episiotomy was divided by the total number of parturients and multiplied by one hundred to calculate the pooled episiotomy practice among African parturients. The pooled effect was investigated using the OR. Furthermore, variables identified as a risk factor for episiotomy in at least three studies were taken into account.

### Publication Bias and Heterogeneity

To check for publication bias, we used Egger's statistical tests and funnel plots. The presence of publication bias was thus declared with a statistical significance of 5%. The *I*^2^-test was also used to determine heterogeneity. When the *I*^2^-test value was 25, 50, and 75%, heterogeneity was classified as mild, moderate, and high, respectively, across the studies.

### Statistical Methods and Analysis

All the extracted data was exported to STATA version 14 software for analysis. Due to the high heterogeneity among the included studies, the random-effects model was used for analysis. To find the source of heterogeneity, we used subgroup analysis based on African regions and meta-regression based on year of publication and sample size. The impact of the retrieved associated factors on the outcome variable was also investigated. Texts, forest plots, and tables were used to illustrate the findings of this systematic review and meta-analysis. The characteristics of the included studies were described using the OR with a 95% CI.

## Result

### Study Search and Selection

**Figure 1:**
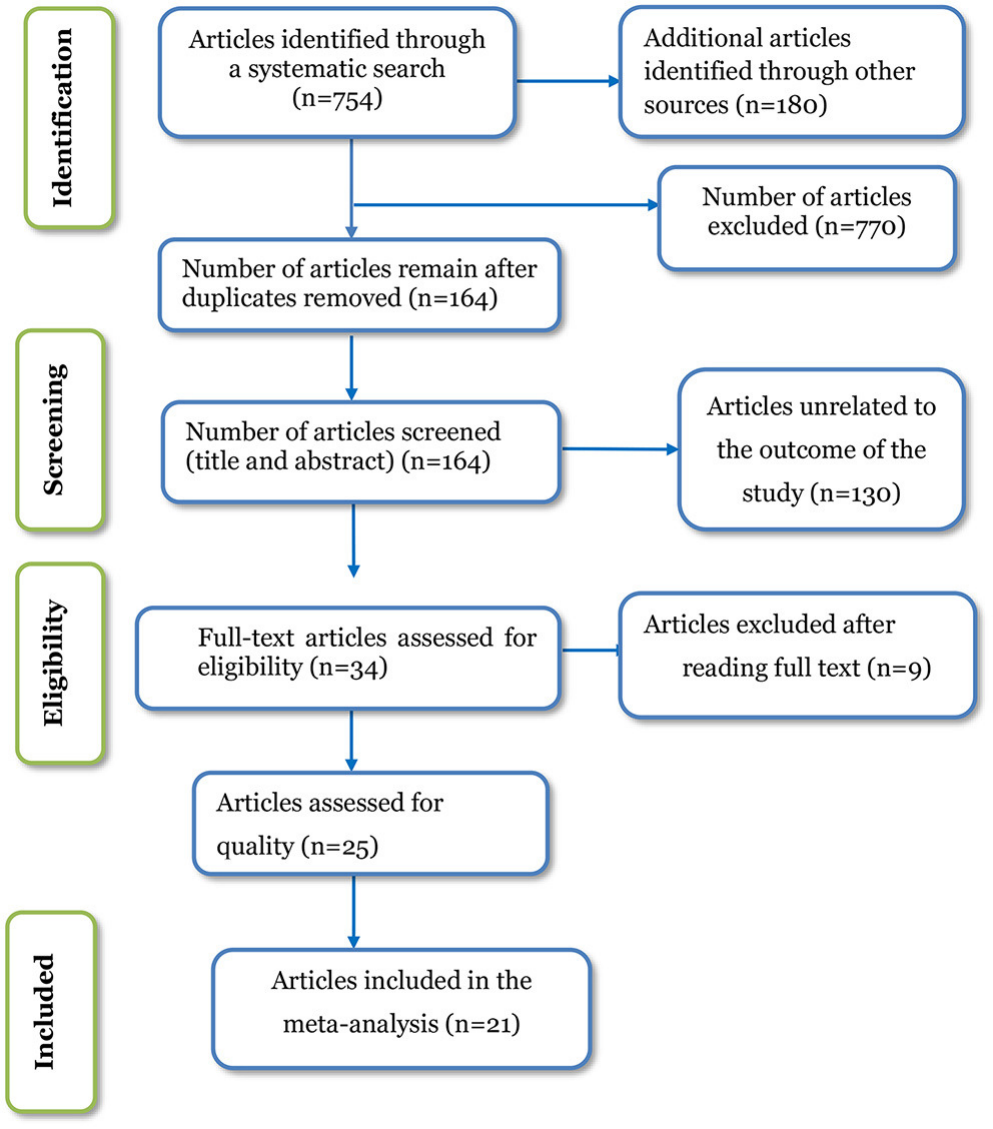
PRISMA flow diagram of included studies in the systematic review and meta-analysis of episiotomy practice and its associated factors in Africa, 2022.

Our search was restricted to articles published in English between January 1, 2000 and December 31, 2021 in the electronic databases PubMed, Hinari, Science Direct, Web of Science, CINAHL, and EMBASE. In addition, Google, Google scholar, and AJOL were used. Through systematic and manual searching, 934 primary articles were found. Due to duplication, 770 articles were removed. The remaining 164 were screened based on their title and abstract, with 130 being eliminated as unrelated to our study. Finally, 34 full-text primary articles were evaluated against eligibility criteria, and 21 were selected for quantitative analysis (Figure 1).

### Study Characteristics

This systematic review and meta-analysis included a total of 21 articles from seven African countries. Eighteen primary studies employed an analytical cross-sectional study design, while the remaining three studies employed a descriptive cross-sectional study design. Regarding the settings in which the studies were conducted eight studies were conducted in Ethiopia (45, 46, 54, 57, 61, 64, 66), eight in Nigeria (40–42, 58, 63, 65, 82), and the remainder of the studies were conducted in the Democratic Republic of the Congo (DRC) (69), Zimbabwe (83), Uganda (55), Ghana (84), and Burkina Faso (85). The majority of the studies, 16 (76.2%), were reported from Eastern and Western African countries. The number of study participants ranged from 249 (55), in a study conducted in Uganda to 12,168 (56) in Nigeria. Out of 40,831 women, episiotomy was practiced on 15,437 of them. The highest prevalence of episiotomy practice was reported in a study from Uganda, at 73% (55), followed by Ethiopia, at 68% (64). On the contrary, the lowest rate of episiotomy, 9.3%, was reported in a study from Nigeria (40). In this meta-analysis, 17 of the 21 studies reported a percentage of episiotomy practice in primiparous and multiparous women (40–42, 45, 46, 53, 54, 56–58, 61, 63–66, 69, 83), while the other four studies did not (55, 82, 84, 85). The mean age of the study participants was not reported in ~66.7% of the studies (40, 46, 55, 56, 58, 63, 64, 66, 69, 82–84). Among the studies that reported the age of the study subjects, the mean age of women for whom episiotomy was performed was 25.57 years (41, 45, 53, 54, 57, 61, 85). The year of publication of the included studies ranged from 2003 to 2021 (Table 1).

**Table 1:** The characteristics of the studies included in the systematic review and meta-analysis.

**References**	**Year**	**Country**	**Region**	**Study design**	**Total**	**Primiparous**		**Multi parous**		**Overall**	**Quality**
					**cases/Total**	**Cases**	**%**	**Cases**	**%**	**(%)**	
					**sample**	**Sample**		**Sample**			
Yemaneh et al. (61)	2015	Ethiopia	East Africa	Cross-Sectional	140/338	95/195	48.7	45/143	31.5	41.4	Low risk
Woretaw et al. (66)	2021	Ethiopia	East Africa	Cross-Sectional	181/410	NR	NR	NR	NR	44.2	Low risk
Kidane et al. (45)	2016	Ethiopia	East Africa	Cross-sectional	144/407	70/140	50.0	74/267	27.7	35.4	Low risk
Worku et al. (57)	2019	Ethiopia	East Africa	Cross-Sectional	134/387	102/158	64.6	32/229	14.0	35.2	Low risk
Beyene et al. (53)	2020	Ethiopia	East Africa	Cross-Sectional	169/411	99/154	64.3	70/257	27.2	41.1	Low risk
Fikadu et al. (64)	2020	Ethiopia	East Africa	Cross-Sectional	272/400	171/212	80.7	101/188	53.7	68.0	Low risk
Tobiaw Tefera and Mekonen (54)	2019	Ethiopia	East Africa	Cross-Sectional	265/405	181/215	86.1	84/190	44.2	65.4	Low risk
Teshome et al. (46)	2020	Ethiopia	East Africa	Cross-Sectional	146/306	118/221	53.4	28/85	32.9	47.7	Low risk
Okeke et al. (58)	2012	Nigeria	West Africa	Cross-Sectional	1201/3,032	624/789	79.1	577/2,243	25.7	39.6	Low risk
Alayande et al. (42)	2012	Nigeria	West Africa	Cross-Sectional	96/280	69/111	62.2	27/169	16.0	34.3	Low risk
Onah and Akani (65)	2004	Nigeria	West Africa	Cross-Sectional	175/433	99/130	76.2	76/303	25.1	40.4	Low risk
Izuka et al. (82)	2014	Nigeria	West Africa	Cross-Sectional	411/662	NR	NR	NR	NR	62.1	Low risk
Chigbu et al. (41)	2008	Nigeria	West Africa	Cross-Sectional	1,877/4,174	1,150/1,277	90.1	727/2,897	25.1	45.0	Low risk
Owa et al. (40)	2015	Nigeria	West Africa	Cross-Sectional	68/728	44/212	20.8	24/516	4.7	9.3	Low risk
Ayyuba et al. (56)	2016	Nigeria	West Africa	Cross-Sectional	5,040/12,168	2,844/3,582	79.4	2,196/8,586	25.6	41.4	Low risk
Enyindah et al. (63)	2007	Nigeria	West Africa	Cross-Sectional	1,846/4,720	972/1,260	77.1	874/3,460	25.3	39.1	Low risk
Pebolo et al. (55)	2019	Uganda	East Africa	Cross-Sectional	181/249	NR	NR	NR	NR	73.0	Low risk
Innocent et al. (69)	2018	DRC	Central Africa	Cross-Sectional	939/1,878	378/492	76.8	561/1,386	40.5	50.0	Low risk
Bergh et al. (83)	2003	Zimbabwe	East Africa	Cross-Sectional	965/3,589	838/1,560	53.8	127/2,029	6.3	27.0	Low risk
Morhe et al. (84)	2004	Ghana	West Africa	Cross-Sectional	374/2,151	268/847	31.6	0/1,304	0	17.4	Low risk
Adama et al. (85)	2018	Burkina Faso	West Africa	Cross-Sectional	813/3,703	NR	NR	NR	NR	22.0	Low risk

*DRC, Democratic Republic of the Congo; NR, Not reported; %, Percentage*.

Regarding the sampling techniques and data collection tools, nine studies employed systematic random sampling methods (45, 46, 53–55, 57, 61, 64, 66), eleven studies employed either retrospective (40–42, 56, 58, 63, 65, 82–84) or prospective (85) analysis of delivery notes, while the remaining one study employed stratified sampling methods (69). Furthermore, pre-tested questionnaires and refined checklists were used to collect data (Supplementary File 5).

### Meta-Analysis

#### Prevalence of Episiotomy Practice

**Figure 2:**
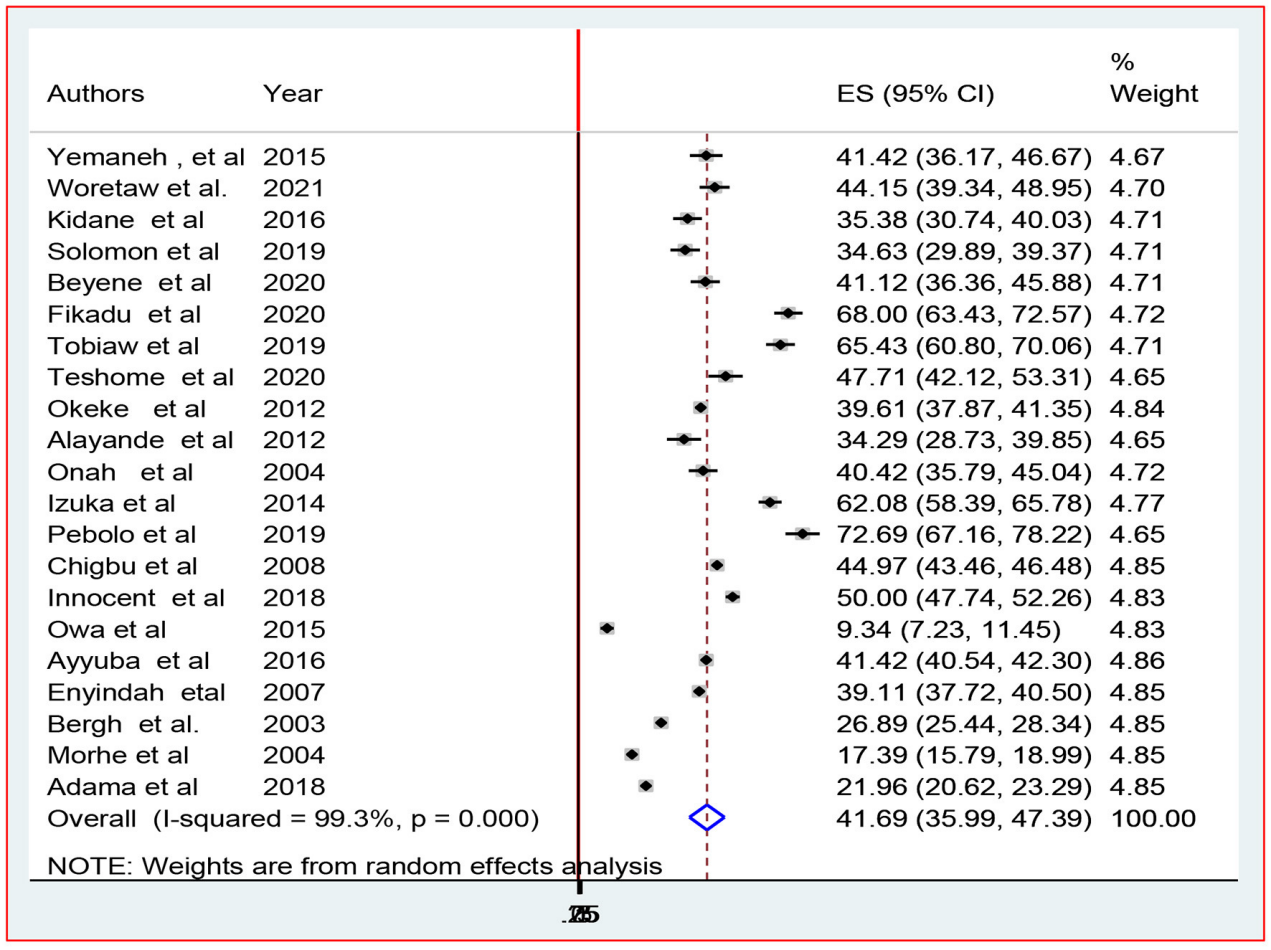
Overall pooled prevalence of episiotomy practice in Africa, 2022.

This systematic review and meta-analysis included 21 studies to estimate the pooled prevalence of episiotomy practice among African parturients who gave birth in health facilities. The heterogeneity (*I*^2^) of the included studies was (*I*^2^ = 99.3%; *P* < 0.001) when using the fixed effect model. Due to the high heterogeneity of the data, we used a random effects model to estimate the pooled prevalence of episiotomy practice, which was 41.7% [95% CI (36.0–47.4)] (Figure 2).

#### Supplementary Analysis

**Figure 3:**
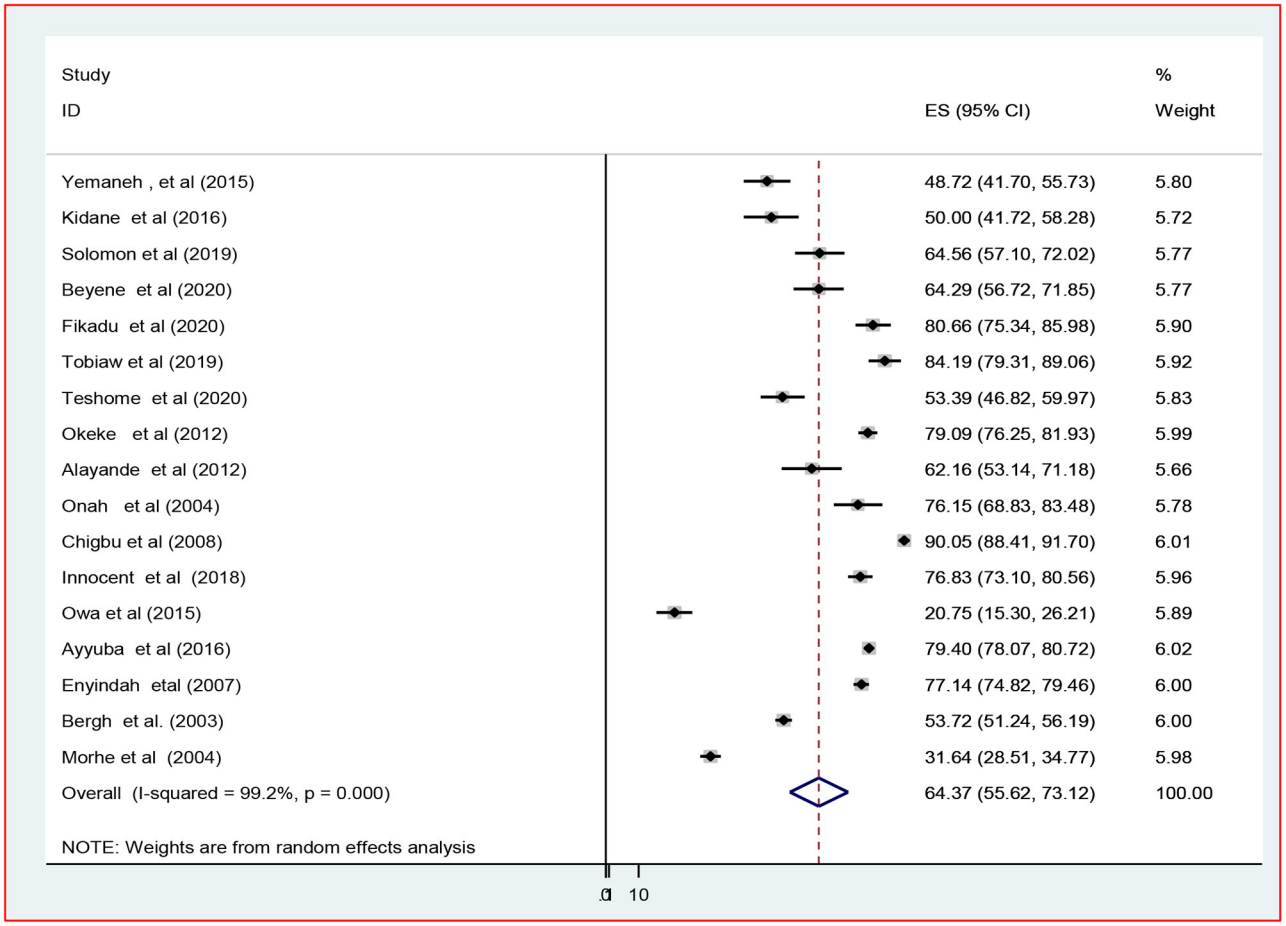
The pooled prevalence of episiotomy practice among primiparous women in Africa, 2022.

**Figure 4:**
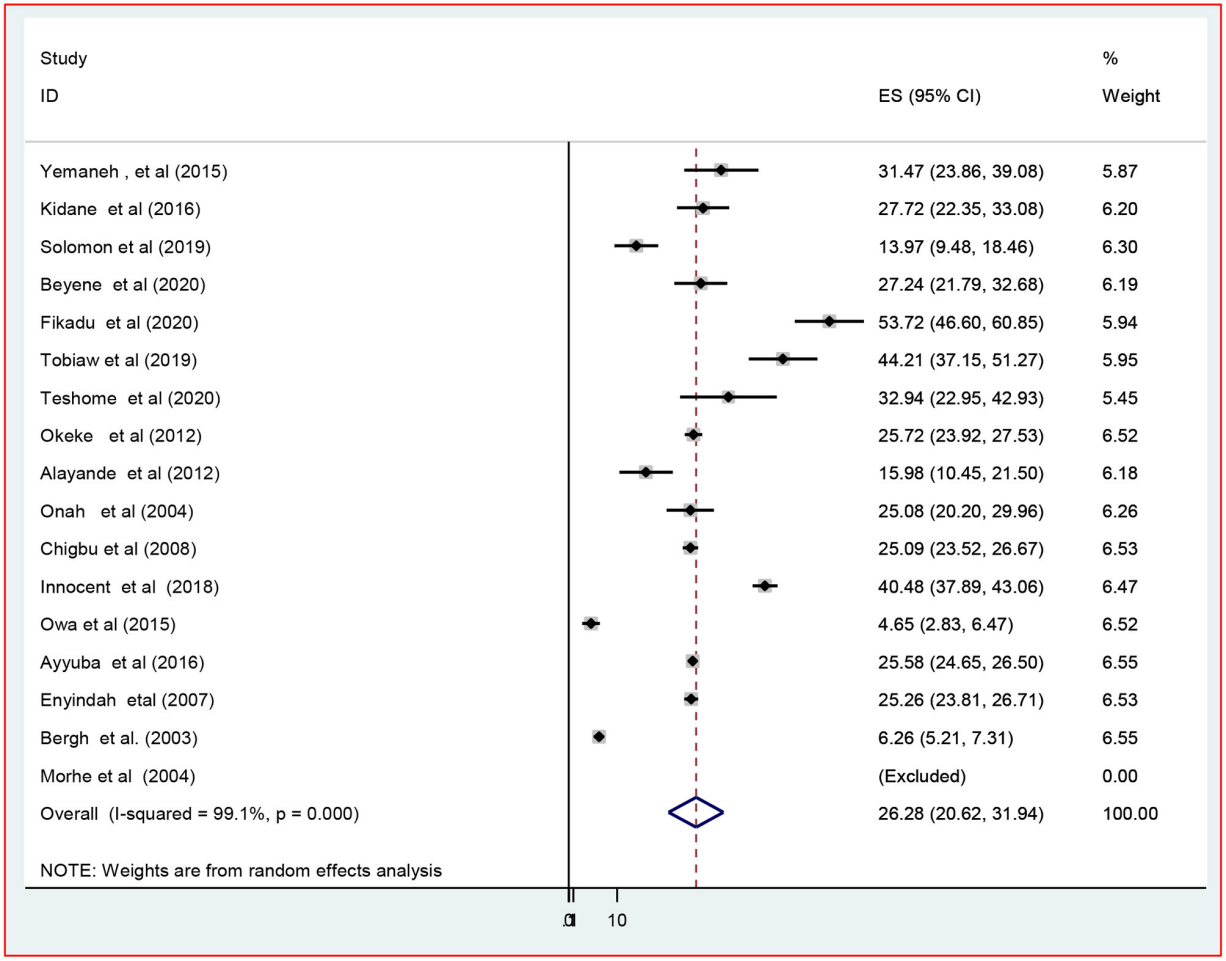
The pooled prevalence of episiotomy practice among multiparous women in Africa, 2022.

In addition, supplementary analysis was performed on 17 of the 21 studies that reported the magnitude of episiotomy in primiparous (*n* = 11,555) and multiparous women (*n* = 24,252) to estimate the percentage of episiotomy practiced. As a result, the combined prevalence of episiotomy among primiparous and multiparous women was 64.4% [(95% CI: 55.6–73.1), *I*^2^ = 99.2%, *P* < 0.001] and 26.3% [(95% CI: 20.6–31.9), *I*^2^ = 99.1%, *P* < 0.001] among primiparous (Figure 3) and multiparous (Figure 4) respectively.

#### Heterogeneity

We used subgroup analysis based on African regions, as well as meta-regression based on year of publication and sample size, to find the source of heterogeneity.

#### Subgroup Analysis by Region

**Figure 5:**
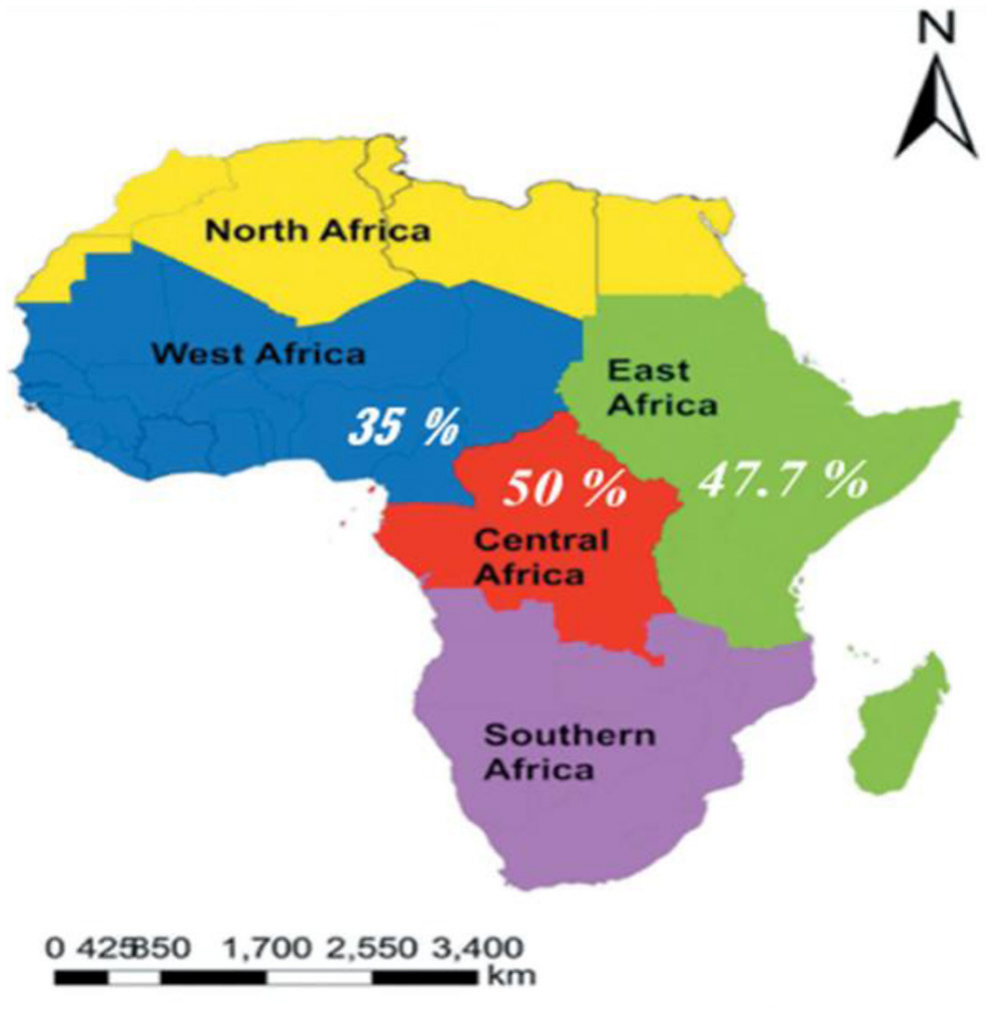
Subgroup analyses on the pooled prevalence of episiotomy practice by African regions, 2022 (source: https://en.wikipedia.org/wiki/List_of_regions_of_Africa).

As shown in Figure 5, a significant variation in episiotomy practice can be seen across the three regions in Africa. Episiotomy was most performed in central Africa 50 % [(95% CI: 47.74–52.26), *I*^2^ = .%, *P* < 0.001] (69). East Africa came in second with 47.7% [(95% CI: 36.55–58.44), *I*^2^ = 98.7 %, p < 0.001] (45, 46, 53, 54, 57, 61, 64, 66, 83). In western African countries, episiotomy was performed in 35.01% [(95% CI: 26.99–43.02), *I*^2^ = 99.6%, *p* < 0.001] (40–42, 56, 58, 63, 65, 82, 84, 85) of parturients. Of the 21 included studies, 10 studies were reprorted from East African countries, while ten studies and one study were reported from westAfrican, and central African countries respectively.

#### Meta Regression

Based on sample size and publication year, we used random-effects meta regression to find the source of heterogeneity at a 5% significance level. As shown in Table 2, these covariates were not found to be the source of heterogeneity.

**Table 2:** Meta regression analysis of factors affecting between study heterogeneity.

**Heterogeneity source**	**Coefficient**	**Standard error**	***T***	***P* > t**	**[95% conf. interval]**	
Year	0.0088525	0.0426002	0.21	0.838	−0.0806471	0.0983522
Sample size	0.0000309	0.0000559	0.55	0.588	−0.0000865	0.0001482

#### Sensitivity Analysis

We used the random-effects model to perform sensitivity analysis to determine the impact of a single study on the overall meta-analysis. The results of the analysis revealed that single study estimates are closer to the combined estimate, implying that a single study has no effect on the final pooled prevalence of episiotomy practice (Table 3).

**Table 3:** Sensitivity analysis of pooled prevalence with each study removed one by one.

**Study omitted**	**Estimate**	**95% [confidence interval]**	
Yemaneh et al. (61)	41.705051	35.847664	47.562439
Woretaw et al. (66)	41.570477	35.714336	47.42662
Kidane et al. (45)	42.004498	36.133785	47.87521
Worku et al. (57)	42.041344	36.172348	47.910336
Beyene et al. (53)	41.720245	35.8564	47.584087
Fikadu et al. (64)	40.381493	34.705494	46.057491
Tobiaw Tefera and Mekonen (54)	40.511047	34.801342	46.220753
Teshome et al. (46)	41.397526	35.557297	47.237751
Okeke et al. (58)	41.805637	35.741482	47.869793
Alayande et al. (42)	42.053123	36.194939	47.911308
Onah and Akani (65)	41.755241	35.888203	47.622284
Izuka et al. (82)	40.66296	34.972534	46.353386
Pebolo et al. (55)	40.173367	34.488853	45.857876
Chigbu et al. (41)	41.530357	35.533012	47.527702
Innocent et al. (69)	41.268299	35.447868	47.08873
Owa et al. (40)	43.314487	37.978573	48.650406
Ayyuba et al. (56)	41.726723	35.287895	48.16555
Enyindah et al. (63)	41.836052	35.640514	48.03159
Bergh et al. (83)	42.452347	36.423828	48.480865
Morhe et al. (84)	42.917702	37.378407	48.456997
Adama et al. (85)	42.694324	36.942516	48.446133
Combined	41.690794	35.994615	47.386973

#### Publication Bias

**Figure 6:**
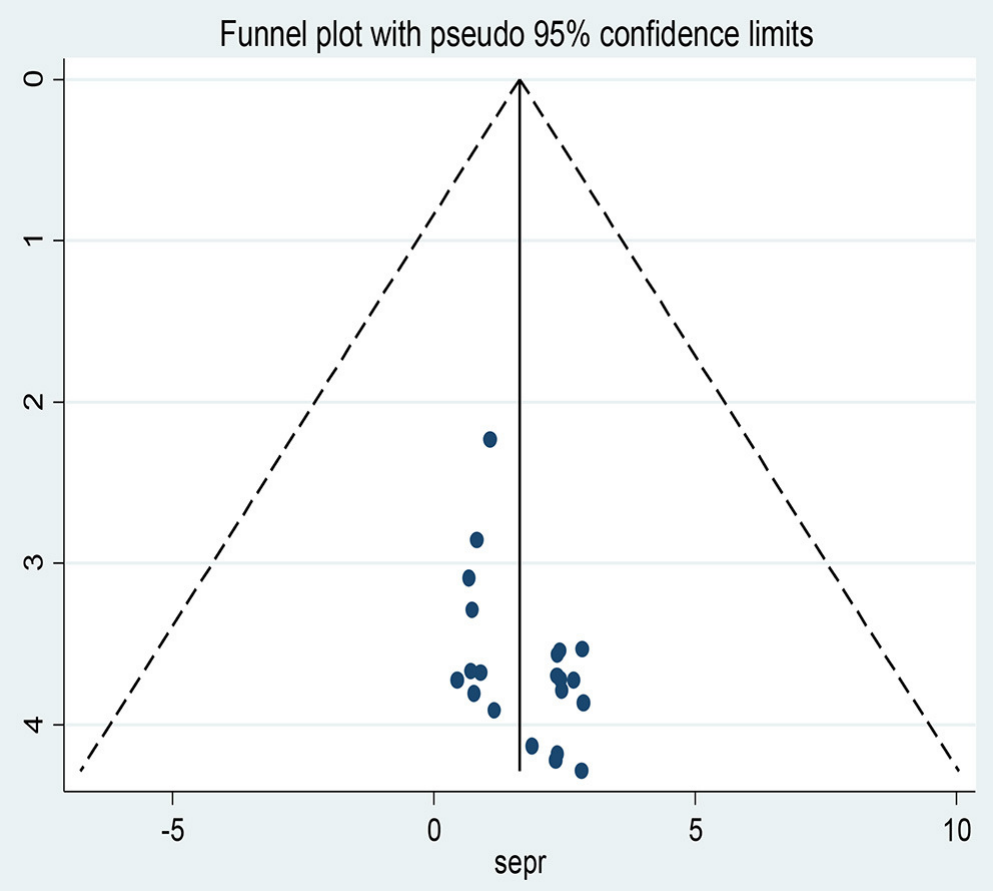
Funnel plots for publication bias for episiotomy practice in Africa, 2022.

All studies fell within the funnel plot based on subjective inspection (Figure 6). Furthermore, neither Egger's linear regression test (*t* = 0.16, *P* = 0.260) nor Begg's rank correlation test (*z* = 0.15, *P* = 0.880) were statistically significant (Table 4).

**Table 4:** Egger's test for small study effects.

**Standard**	**Coefficient**	**Standard**	***t***	***P* > |t|**	**[95% confidence interval]**	
**effect**		**error**				
Slope	30.73632	5.047684	6.09	0.000	20.1714	41.30125
Bias	5.587953	4.809744	1.16	0.260	−4.478956	15.65486

#### Factors Associated With Episiotomy Practice in Africa

The pooled odds ratio was used to identify factors linked to episiotomy practice, and the association with the outcome variable was declared at a 5% significant level. As a result, eight variables were extracted from at least three studies. Six variables were found to be predictors of episiotomy performance: birth attendant, mode of delivery, oxytocin use, prolonged second stage of labor, birth weight, and parity.

This study revealed that primiparous women had 6.78 times more likelihood of incurring an episiotomy as compared to multiparous women [OR: 6.796 (95% CI (4.862–9.498)), *P* < 0.001, *I*^2^: 95.1%].Furthermore, episiotomy was 3.7 times more likely to be practiced when medical doctors attended the delivery compared to midwives [OR: 3.675 (95% CI (2.034–6.640)), *P* < 0.001, *I*^2^: 72.6%]. In this study, the second stage of labor delayed for more than 2 h was 5.5 times more likely to end up with an episiotomy [OR: 5.539 (95% CI (4.252–7.199)), *P* < 0.001, *I*^2^: 0.0%].

In the current review, laboring mothers who were given oxytocin for labor augmentation were 4.21 times more likely to undergo episiotomy when compared to their counterparts [OR: 4.207 (95% CI (3.100–5.709)), *P* < 0.001, *I*^2^: 0.0%]. Regarding the mode of delivery, mothers whose labor was assisted by instrumental vaginal delivery were 5.58 times more likely to undergo episiotomy compared to those delivered by spontaneous vertex delivery [OR: 5.578 (95% CI (4.285–7.260)), *P* < 0.001, *I*^2^: 65.1%]. Furthermore, the findings from the current review revealed that the odds of episiotomy practice were 5.32 times more likely when the fetal birth weight was ≥4,000 g [OR: 5.32 (95% CI (2.738–10.339)), *P* < 0.001, *I*^2^: 95.1%] compared to the normal birth weight (Table 5).

**Table 5:** Factors associated with episiotomy practice in Africa, 2022.

**Variables**	**Comparison**	**Number**	**Sample**	**Pooled**	***P*-value**	***I^**2**^***	**Heterogeneity**	**Egger**
		**of studies**	**size**	**OR (95% CI)**		**(%)**	**test**	**test**
Age of the mother (41, 42, 45, 46, 65)	<35/≥35	5	5,895	0.879 (0.104–7.420)	0.906	94.7	<0.001	0.757
Birth attendant (40, 57, 61)	Physicians vs. midwives	3	1,447	3.675 (2.034–6.640)	<0.001*	72.6	0.026	0.764
Gestational age (45, 55, 63)	≥37 vs. <37 wks	3	5,347	1.232 (0.604–2.513)	0.565	72.6	0.026	0.727
Mode of delivery (40–42, 53, 54, 56, 58, 61, 63, 66)	None SVD vs. SVD	10	26,643	5.578 (4.285–7.260)	<0.001*	65.1	0.002	0.15
Oxytocin use (45, 53, 66)	Yes vs. No	3	1,228	4.207 (3.100–5.709)	<0.001*	0.0	0.488	0.559
Prolonged second labor (46, 53–55, 57)	Yes vs. No	5	1,750	5.533 (4.252–7.199)	<0.001*	0.0	0.456	0.104
Birth weight (42, 53, 54, 56–58)	Macrosomia vs. normal	6	16,677	5.320 (2.738–10.339)	<0.001*	78.3	<0.001	0.084
Parity (40–42, 45, 46, 53, 56–58, 61, 63–65)	Primiparity vs. multiparty	13	27,778	6.796 (4.862–9.498)	<0.001*	95.1	<0.001	0.051

SVD, Spontaneous vertex delivery; vs, versus; OR, Odds Ratio; CI, Confidence Interval;* a significant level at 5%.

## Discussion

The aim of this meta-analysis was to find out how common episiotomy was and what factors were linked to it. This review included twenty-one studies in order to summarize the extent of episiotomy use and identify associated factors among African women who gave birth in public health facilities. These results have been obtained from research conducted in a number of African countries. The routine use of episiotomy, according to researchers, increases the risk of perineal trauma (27). All international organizations, including the WHO, agree with the body of evidence that routine episiotomy has no place in the modern era of advanced maternal care (75). Furthermore, the 2006 ACOG bulletin did not recommend the routine use of episiotomy (3).

Nonetheless, a remarkable spectrum of episiotomy practice has been observed among countries around the globe (82). The current review found that the pooled prevalence of episiotomy practices among laboring mothers in Africa was 41.7 [95% CI (36.0–47.4)] for all vaginal deliveries. There is a wide difference in episiotomy practice from region to region within the African continent. The sample size, year of publication, and settings where the studies were conducted might have contributed to a high and uneven spectrum of episiotomy. Furthermore, such disparities may indicate a lack of evidence-based standardized policy, training, and practice across the continent. Another possible explanation for the variation in episiotomy practices among African countries could be the preference to employ episiotomy frequently because of the simultaneous belief that allowing even minor perineal tears is more cumbersome than repair when an episiotomy cut is performed.

In the current review, the rate of episiotomy for all vaginal deliveries is by far higher than previous studies carried out in Denmark, 4.9% (20), Sweden, 6.6% (20), Nigeria, 7% (40), the United States in 2011, 9.4% (20), Ghana, 17% (84), United Kingdom (UK) in 2011/2012, 15.20% (20), Burkina Faso, 21% (85), in rural Zimbabwe, 26% (83), Hong Kong, 27% (86), Vietnamese-born women living in Australia, 29.9% (30), Slovania, 31.3% in 2013 (87), and 14.3% in France (88). A significant and continuing decline in the rate of episiotomy practices in some countries over the years may be attributed to the adoption of clinical practice guidelines advocating the policy of restricted episiotomy use. Clesse et al. (88), for instance, found a remarkable and persistent decline in the episiotomy figure 7.3 of percentage points and a rate of change of 34% from 2013.

Similarly, the episiotomy rate in all vaginal deliveries in the United States fell from 60.9% in 1979 to 24.5% in 2004 (89), and the rate in Hong Kong fell from 73% in 2003 to 27% in 2008 (86). In contrast, the pooled estimate for episiotomy practice, in all vaginal deliveries, in this meta-analysis was lower than in a study conducted in India, where 63.4% (90), Oman, 66% (27), Portugal, 72.9% in 2010 (20), Mexico City, 77.2% (25), Turkey, 93.3% (91), Phnom Penh, Cambodia, 94.5% (28), and Taiwan, 100% (23), which may suggest liberal use in these countries.

This systematic review and meta-analysis also identified potential determinants of episiotomy practices among parturients in Afrika. Thus, primiparity was found to be significantly associated with the use of episiotomy, which is supported by the findings of other previous studies (30, 68). Another study also found that primiparas were more likely to undergo episiotomy than multiparas (28). The results of this review are also in line with those of previous studies conducted in Ethiopia (45, 46, 53, 57, 61, 64), Nigeria (40–42, 56, 58, 63, 65), Brazil (92), Vietnam (93), Iran (67), Saudi Arabia (94), Latin America (59), France (68), East African migrants in Australia (95), Taiwan (96), and Vietnam born women in Australia (30). The potential explanation may be that episiotomies are thought to speed up the second stage of labor and reduce the risk of spontaneous perineal tears, but such perceptions among obstetricians or midwives have not been supported by evidence.

Fetal macrosomia is common in obstetrics with problems in both the mother and newborn. The current review also showed that newborn birth weight ≥4,000 g was another risk factor associated with the practice of episiotomy compared with normal birth weight, which coincides with the findings reported in studies carried out in Ethiopia (53, 54, 57), France (51), and Nigeria (42, 56, 82). Other than newborns', fetal macrosomia causes maternal complications during delivery, such as 3rd or 4th degree perineal tears (97, 98). Rates of episiotomy, and other morbidities and mortality associated with predicted macrosomia could be reduced by cesarean deliveries (99). However, when such prenatal screening is not available as in underdeveloped countries, it contributes to high magnitudes of episiotomy.

Statistical analysis of this result also indicates that a protracted second stage of labor is among the important risk factors positively associated with episiotomy. This finding is also supported by studies conducted in Iran (67), Spain (100), and Brazil (92). When mothers exert themselves in labor for more than 2 h, they usually become exhausted. Moreover, inadequate provision of maternal support will also result in prolonged labor. This time, the attending healthcare professional is forced to perform an episiotomy cut to alleviate or reduce morbidity to the fetus (53).

In present study, laboring mothers who had used oxytocin for the induction or augmentation of labor had higher odds of being exposed to episiotomy than their counterparts. Our results concur with the findings in Iran (67), Brazil (92), Vietnam (30), and Latin America (32). Similarly this review is congruent with a study conducted in Shroud City, northeast Iran (67). The potential explanation could be due to oxytocin induced uterine hyper stimulation which in turn which may affect the normal beat to beat variability during labor resulting in non-reassuring fetal heart rate patterns. In such cases episiotomy is usually performed for expeditious delivery of the baby in an attempt to shorten labor time.

None spontaneous vertex deliveries (vacuum-assisted, forceps, and assisted breech deliveries) were another risk factor for episiotomy practice in laboring mothers compared with spontaneous vertex delivery and it is in line with previous studies conducted in other settings (32, 37, 67, 94). Such a correlation may arise from tertiary hospitals' endeavors to handle abnormal labor, complex and advanced maternity care. Therefore, doctors and midwives may perform an episiotomy to decrease perineal tears in such a situation.

The other finding from this study is that deliveries attended by doctors were positively associated with episiotomy practice compared with deliveries attended by midwives. Similar findings have been observed in other settings (40, 57, 61). One of the reasons might be that abnormal labors are frequently attended by medical doctors and, hence, episiotomies are performed liberally to support and assist the labor process with forceps or vacuum delivery.

### Limitations of This Study

The limitations of this systematic review have been acknowledged. Some studies did not contain sufficient predictor variables to adequately determine the degree of prediction. However, attempts were made to include all other potential variables across the identified databases. Furthermore, in this review, the study method used in all included articles was a cross-sectional design. As a result, the outcome variable might be affected by other confounding variables, which would decrease the power of the study and the causal conclusion between episiotomy and its associated factors. In the current meta-analysis, all included studies were conducted in African countries in three regions: Eastern Africa, Central Africa, and Western Africa. Therefore, it might lack continental representativeness because no information was found in the northern and southern regions of the African continent. However, the maternal health care and health care facilities in these regions are not different from those in other regions of the continent. Furthermore, the results of this review should be interpreted cautiously as there is significant heterogeneity in pooled effect estimates.

### Strength of This Study

The protocol for this study has been registered. More than seven online databases were searched to avoid missing published studies, including articles published in African journals. In addition, a manual search was performed to retrieve the article using Google Scholar. During the selection of articles, the PRISMA guidelines were strictly followed, and the articles were closely assessed for their quality using the newly amended JBI critical appraisal tool. Furthermore, we used broader inclusion criteria to include articles published from 2000 to 2021GC. In studies that reported percent of episiotomy in both primiparous and multiparous parturients, additional analysis was performed. A sensitivity analysis was also carried out.

## Conclusion

The pooled random effect meta-analysis revealed that the prevalence of episiotomy practice among laboring mothers in Africa was high when compared to existing global recommendations, including those from the WHO. Furthermore, primiparity, macrosomia, prolonged second stage of labor, instrument assisted vaginal deliveries, augmented or induced labor using oxytocin, and deliveries attended by medical doctors were independent predictors of episiotomy practice in African health facilities.

## Recommendations

As a result, we recommend that African countries adopt a restrictive episiotomy policy to lower their rates and limit morbidity associated with injudicious episiotomy practice. To reduce the risks associated with macrosomia, prenatal screening with obstetric ultrasound and cesarean section delivery should be encouraged. Countries should either follow international guidelines like WHO and ACOG recommendations or create their own protocols. To change current beliefs about episiotomy in primiparous women, more in-service training for midwives and obstetricians is required. Episiotomy should only be performed when there is a clear indication or when evidence supports it.

## Data Availability Statement

The original contributions presented in the study are included in the article/Supplementary Material, further inquiries can be directed to the corresponding author.

## Author Contributions

BW and MO involved in selection of study, data extraction, quality assessment, statistical analysis, results interpretation and writing the initial, and final drafts of the manuscript. EB, LT, TB, and HA were involved in data extraction, quality assessment, statistical analysis, and writing drafts of the manuscript. All authors proofread and approved the final manuscript.

## Conflict of Interest

The authors declare that the research was conducted in the absence of any commercial or financial relationships that could be construed as a potential conflict of interest.

## Publisher's Note

All claims expressed in this article are solely those of the authors and do not necessarily represent those of their affiliated organizations, or those of the publisher, the editors and the reviewers. Any product that may be evaluated in this article, or claim that may be made by its manufacturer, is not guaranteed or endorsed by the publisher.

## Abbreviations

ACOG: American College of Obstetricians and Gynecologists
CI: Confidence Interval
JBI: Johanna Briggs Institute
OR: Odds Ratio
PRISMA: Preferred Reporting Items for Systematic Reviews and Meta-Analyses
USA: United States of America
WHO: World Health Organization.

## References

[1] Thacker S Banta H. Benefits and risks of episiotomy: an interpretive review of the English language literature, 1860-1980. Obstet Gynecol Survey. (2003) 38:322–38. 10.1097/00006254-198306000-00003 6346168

[2] Kalis V Laine K de Leeuw JW Ismail KM Tincello DG. Classification of episiotomy: towards a standardisation of terminology. BJOG. (2012) 119:522–6. 10.1111/j.1471-0528.2011.03268.x 22304364

[3] American College of Obstetricians-Gynecologists. ACOG practice bulletin. episiotomy. Clinical management guidelines for obstetrician-gynecologists. number 71, April 2006. Obstet Gynecol. (2006) 107:957–62. 16582142

[4] Löwenstein L Drugan A Gonen R Itskovitz-Eldor J Bardicef M Jakobi P. Episiotomy: beliefs, practice and the impact of educational intervention. Euro J Obstetr Gynecol Reprod Biol. (2005) 123:179–82. 10.1016/j.ejogrb.2005.04.006 15913881

[5] Frigerio M Mastrolia SA Spelzini F Manodoro S Yohay D Weintraub AY. Long-term effects of episiotomy on urinary incontinence and pelvic organ prolapse: a systematic review. Arch Gynecol Obstet. (2019) 299:317–25. 10.1007/s00404-018-5009-9 30564925

[6] Cassado Garriga J Carmona Ruiz A Pessarrodona Isern A Rodriguez Carballeira M Esteve Serena E Garcia Manau P . Impact of episiotomy on the urogenital hiatus using transperineal ultrasound. Neurourol Urodyn. (2018) 37:434–9. 10.1002/nau.23322 28598517

[7] Dillon SJ Nelson DB Spong CY McIntire DD Leveno KJ. Episiotomy: evolution of a common obstetric practice at a public hospital. Am J Perinatol. (2021) 1620–35. 10.1055/s-0041-1739410 34856609

[8] Jiang H Qian X Carroli G Garner P. Selective versus routine use of episiotomy for vaginal birth. Cochrane Database Syst Rev. (2017) 2:Cd000081. 10.1002/14651858.CD000081.pub3 28176333 PMC5449575

[9] Dogan B Gün I Özdamar Ö Yilmaz A Muhçu M. Long-term impacts of vaginal birth with mediolateral episiotomy on sexual and pelvic dysfunction and perineal pain. J Mater Fetal Neonatal Med. (2017) 30:457–60. 10.1080/14767058.2016.1174998 27112425

[10] Ye J Chen Y Yang H Chen Q Huang Y Zhao J . A nationwide cross-sectional survey of episiotomy practice in China. Lancet Reg Health West Pac. (2022) 19:100345. 10.1016/j.lanwpc.2021.100345 35024669 PMC8671730

[11] Steiner N Weintraub AY Wiznitzer A Sergienko R Sheiner E. Episiotomy: the final cut? Arch Gynecol Obstet. (2012) 286:1369–73. 10.1007/s00404-012-2460-x 22810620

[12] Forey P-L Lallemant M Bourtembourg-Matras A Eckman-Lacroix A Ramanah R Riethmuller D . Impact of a selective use of episiotomy combined with Couder's maneuver for the perineal protection. Arch Gynecol Obstet. (2020) 302:77–83. 10.1007/s00404-020-05572-9 32388778

[13] Cluett ER Burns E Cuthbert A. Immersion in water during labour and birth. Cochrane Database Syst Rev. (2018) 5:CD000111. 10.1002/14651858.CD000111.pub4 29768662 PMC6494420

[14] Lathrop A Bonsack CF Haas DM. Women's experiences with water birth: a matched groups prospective study. Birth. (2018) 45:416–23. 10.1111/birt.12362 29900579

[15] Aughey HK Jardine J Moitt N Fearon K Hawdon J Pasupathy D . Waterbirth: a national retrospective cohort study of factors associated with its use in 46,088 women in England. Res Square. (2020) 2–9. 10.21203/rs.3.rs-93490/v133771115

[16] Zaami S Stark M Beck R Malvasi A Marinelli E. Does episiotomy always equate violence in obstetrics? Routine and selective episiotomy in obstetric practice and legal questions. Eur Rev Med Pharmacol Sci. (2019) 23:1847–54. 10.26355/eurrev_201903_17219 30915726

[17] Zaami S Zupi E Lazzeri L Centini G Stark M Malvasi A . Episiotomy: a medicolegal vicious cycle. Panminerva Med. (2020) 63:224–31. 10.23736/S0031-0808.20.03946-4 32414232

[18] Olusanya BO Teeple S Kassebaum NJ. The contribution of neonatal jaundice to global child mortality: findings from the GBD 2016 study. Pediatrics. (2018) 141:e20171471. 10.1542/peds.2017-1471 29305393

[19] United Nations Department Department of Economics and Social Affairs Population Division. World Population Prospects: The 2019 Revision. Volume 1. Comprehensive Tables. United Nations, Department of Economics and Social Affairs, Population Division, New York (2019).

[20] Clesse C Lighezzolo-Alnot J De Lavergne S Hamlin S Scheffler M. Statistical trends of episiotomy around the world: comparative systematic review of changing practices. Health Care Women Int. (2018) 39:644–62. 10.1080/07399332.2018.1445253 29509098

[21] Danilack VA Nunes AP Phipps MG. Unexpected complications of low-risk pregnancies in the United States. Am J Obstet Gynecol. (2015) 212:809. e1–809. e6. 10.1016/j.ajog.2015.03.038 26042957 PMC4728153

[22] Chandraharan E. Intrapartum care: an urgent need to question historical practices and ‘non-evidence'-based, illogical foetal monitoring guidelines to avoid patient harm. J Pat Saf Risk Manage. (2019) 24:210–7. 10.1177/2516043519878583

[23] Graham ID Carroli G Davies C Medves JM. Episiotomy rates around the world: an update. Birth. (2005) 32:219–23. 10.1111/j.0730-7659.2005.00373.x 16128977

[24] Friedman AM Ananth CV Prendergast E D'Alton ME Wright JD. Variation in and factors associated with use of episiotomy. JAMA. (2015) 313:197–9. 10.1001/jama.2014.14774 25585333

[25] Garcia-Cerde R Torres-Pereda P Olvera-Garcia M Hulme J. Health care workers' perceptions of episiotomy in the era of respectful maternity care: a qualitative study of an obstetric training program in Mexico. BMC Pregnancy Childbirth. (2021) 21:549. 10.1186/s12884-021-04022-x 34384395 PMC8359587

[26] Seijmonsbergen-Schermers A Geerts C Prins M Van Diem M Klomp T Lagro-Janssen A . The use of episiotomy in a low-risk population in the Netherlands: a secondary analysis. Birth. (2013) 40:247–55. 10.1111/birt.12060 24344705

[27] Al-Ghammari K Al-Riyami Z Al-Moqbali M Al-Marjabi F Al-Mahrouqi B Al-Khatri A . Predictors of routine episiotomy in primigravida women in Oman. Appl Nurs Res. (2016) 29:131–5. 10.1016/j.apnr.2015.05.002 26856503

[28] Schantz C Sim KL Ly EM Barennes H Sudaroth S Goyet S. Reasons for routine episiotomy: a mixed-methods study in a large maternity hospital in phnom Penh, Cambodia. Reprod Health Matters. (2015) 23:68–77. 10.1016/j.rhm.2015.06.012 26278834

[29] Lam K Wong H Pun T. The practice of episiotomy in public hospitals in Hong Kong. Hong Kong Med J. (2006) 12:94. 16603774

[30] Trinh AT Khambalia A Ampt A Morris JM Roberts CL. Episiotomy rate in vietnamese-born women in Australia: support for a change in obstetric practice in Viet Nam. Bull World Health Organ. (2013) 91:350–6. 10.2471/BLT.12.114314 23678198 PMC3646354

[31] Hartmann K Viswanathan M Palmieri R Gartlehner G Thorp J Lohr KN. Outcomes of routine episiotomy: a systematic review. JAMA. (2005) 293:2141–8. 10.1001/jama.293.17.2141 15870418

[32] Althabe F Belizán JM Bergel E. Episiotomy rates in primiparous women in Latin America: hospital based descriptive study. BMJ. (2002) 324:945–6. 10.1136/bmj.324.7343.945 11964339 PMC102327

[33] Lansky S Friche AAL Silva AAM Campos D Bittencourt SDA Carvalho ML . Birth in Brazil survey: neonatal mortality, pregnancy and childbirth quality of care. Cad Saude Publica. (2014) 30:S192–207. 10.1590/0102-311X00133213 25167179

[34] Cunha CMP Katz L Lemos A Amorim MKnowledge M. Attitude and practice of brazilian obstetricians regarding episiotomy. Rev Bras Ginecol Obstet. (2019) 41:636–46. 10.1055/s-0039-3400314 31745956 PMC10316827

[35] Räisänen S Vehviläinen-Julkunen K Gisler M Heinonen S. A population-based register study to determine indications for episiotomy in Finland. Int J Gynecol Obstet. (2014) 115:26–30. 10.1016/j.ijgo.2011.05.008 21767841

[36] Clesse C Lighezzolo-Alnot J De Lavergne S Hamlin S Scheffler M. Factors related to episiotomy practice: an evidence-based medicine systematic review. J Obstet Gynaecol. (2019) 39:737–47. 10.1080/01443615.2019.1581741 31020867

[37] de Carvalho CCM Souza ASR Moraes Filho OB. Prevalence and factors associated with practice of episiotomy at a maternity school in Recife, Pernambuco, Brazil. Rev Assoc Med Bras. (2010) 56:333–9. 10.1590/S0104-42302010000300020 20676543

[38] da Silva FM de Oliveira SM Bick D Osava RH Tuesta EF Riesco ML. Risk factors for birth-related perineal trauma: a cross-sectional study in a birth centre. J Clin Nurs. (2012) 21:2209–18. 10.1111/j.1365-2702.2012.04133.x 22646921

[39] Ezegwui H Ikeako L Ogbuefi F. Obstetric outcome of teenage pregnancies at a tertiary hospital in Enugu, Nigeria. Niger J Clin Pract. (2012) 15:147–50. 10.4103/1119-3077.97289 22718161

[40] Owa O Eniowo A Ilesanmi O. Factors associated with episiotomy among parturients delivering in a tertiary care centre in Nigeria. Int J Res Med Sci. (2015) 3:836. 10.5455/2320-6012.ijrms20150403

[41] Chigbu B Onwere S Aluka C Kamanu C Adibe E. Factors influencing the use of episiotomy during vaginal delivery in South Eastern Nigeria. East Afr Med J. (2008) 85:240–3. 10.4314/eamj.v85i5.9618 18814534

[42] Alayande BT Amole IO Akin D. Relative frequency and predictors of episiotomy in Ogbomoso, Nigeria. Internet J Med Update. (2012) 7:41–4. Available online at: http://www.akspublication.com/ijmu

[43] Demirci O Yilmaz E Tosun Ö Kumru P Arinkan A Mahmutoglu D . Effect of young maternal age on obstetric and perinatal outcomes: results from the tertiary center in Turkey. Balkan Med J. (2016) 33:344–9. 10.5152/balkanmedj.2015.150364 27308080 PMC4898995

[44] Kozhimannil KB Karaca-Mandic P Blauer-Peterson CJ Shah NT Snowden JM. Uptake and utilization of practice guidelines in hospitals in the United States: the case of routine episiotomy. Joint Comm J Qual Pat Saf. (2017) 43:41–8. 10.1016/j.jcjq.2016.10.002 28334585

[45] Kidane GG Gebrehiwot H Abay M Darie Getachew TW. Episiotomy practice and its associated factors among mothers who gave birth vaginally at public health institutions of Shire Town, Northern Ethiopia. Res Rev J Health Prof. (2016) 6:1–8.

[46] Teshome Y Mekonen M Sisay T Chala G Mengistu A Shewasinad S . Prevalence of episiotomy and its associated factors in university of gondar comprehensive specialized referral hospital: a retrospective study from Ethiopia. Am J Life Sci. (2020) 8:9. 10.11648/j.ajls.20200801.12

[47] Wu LC Malhotra R Lie D Tan TC Østbye T. Risk factors and midwife-reported reasons for episiotomy in women undergoing normal vaginal delivery. Arch Gynecol Obstet. (2014) 288:1249–56. 10.1007/s00404-013-2897-6 23708390

[48] Cromi A Bonzini M Uccella S Serati M Bogani G Pozzo N . Provider contribution to an episiotomy risk model. J Mater Fetal Neonatal Med. (2015) 28:2201–6. 10.3109/14767058.2014.982087 25380033

[49] Shmueli M Gabbay Benziv R Hiersch L Ashwal E Aviram R Yogev Y . Episiotomy–risk factors and outcomes. J Mater Fetal Neonatal Med. (2017) 30:251–6. 10.3109/14767058.2016.1169527 27018243

[50] Macleod M Strachan B Bahl R Howarth L Goyder K Van de Venne M . A prospective cohort study of maternal and neonatal morbidity in relation to use of episiotomy at operative vaginal delivery. BJOG. (2008) 115:1688–94. 10.1111/j.1471-0528.2008.01961.x 19035943

[51] Koskas M Caillod A Fauconnier A Bader G. Maternal and neonatal consequences induced by the French recommendations for episiotomy practice. Monocentric study about 5409 vaginal deliveries. Gynecol Obstet Fertil. (2009) 37:697–702. 10.1016/j.gyobfe.2009.06.003 19682940

[52] Giannella L Mfuta K Pedroni D Delrio E Venuta A Bergamini E . Delays in the delivery room of a primary maternity unit: a retrospective analysis of obstetric outcomes. J Mater Fetal Neonatal Med. (2013) 26:593–7. 10.3109/14767058.2012.745500 23126633

[53] Beyene F Nigussie AA Limenih SK Tesfu AA Wudineh KG. Factors associated with episiotomy practices in Bahirdar city, Ethiopia: a cross-sectional study. Risk Manag Healthc Policy. (2020) 13:2281–9. 10.2147/RMHP.S277657 33122956 PMC7591097

[54] Tobiaw Tefera MBK Mekonen T. Prevalence of episiotomy and factors associated with practice of episiotomy at Saint Paul's hospital millennium medical college: a cross sectional study. Ethiop J Reproduct Health. (2019) 11:1–8.

[55] Pebolo F Judith A Kabonge Dan K. Prevalence and factors associated with episiotomy practice among primiparous women in mulago national referral hospital Uganda. Int J Preg Child Birth. (2019) 5:197–201. 10.15406/ipcb.2019.05.00176

[56] Ayyuba R Garba I Ozegya M Abubakar I. Episiotomy at aminu kano teaching hospital, Kano, Nigeria: a 3-year review. Arch Int Surg. (2016) 6:17. 10.4103/2278-9596.187202

[57] Worku SA Mitku YM Getahun SA. Episiotomy-practice-and-its-associated-factor-among-women-who-gave-birth-at-public-health-institutions-of-akaki-kality. Clin Mother Child Health. (2019) 16:1–6. 10.4172/2090-7214.1000318

[58] Okeke T Ugwu E Okezie O Enwereji J Ezenyeaku C Ikeako L. Trends and determinants of episiotomy at the University of nigeria teaching hospital (UNTH), Enugu, Nigeria. Niger J Med. (2012) 21:304–7. Available online at: https://www.ajol.info/index.php/njm/article/view/91062 23304925

[59] Ballesteros-Meseguer C Carrillo-García C Meseguer-de-Pedro M Canteras-Jordana M Martínez-Roche M. Episiotomy and its relationship to various clinical variables that influence its performance. Rev Lat Am Enfermagem. (2016) 24:e2793. 10.1590/1518-8345.0334.2686 27224064 PMC4877173

[60] Lesieur E Blanc J Loundou A Dubuc M Bretelle F. Can the rate of episiotomy still be lowered? Status update in PACA region (south of France). Gynecol Obstet Fertil Senol. (2017) 45:146–51. 10.1016/j.gofs.2017.01.007 28682756

[61] Yemaneh Y Sahile E Alehegn A Girma A Robles C. Assessment of the proportion and associated factors of episiotomy at public health institutions of Axum town, Tigray Region, North Ethiopia. Crit Care Obst Gyne. (2015) 11:1–7. 10.21767/2471-9803.1000152

[62] Chuilon A Le Ray C Prunet C Blondel B. Episiotomy in France in 2010: Variations according to obstetrical context and place of birth. J Gynecol Obstet Biol Reprod. (2016) 45:691–700. 10.1016/j.jgyn.2015.10.005 26996239

[63] Enyindah C Fiebai P Anya S Okpani A. Episiotomy and perineal trauma prevalence and obstetric risk factors in Port Harcourt, Nigeria. Niger J Med. (2007) 16:242–5. 17937162

[64] Fikadu K Boti N Tadesse B Mesele D Aschenaki E Toka E . Magnitude of episiotomy and associated factors among mothers who give birth in arba minch general hospital, southern ethiopia: observation-based cross-sectional study. J Pregnancy. (2020) 2020:8395142. 10.1155/2020/8395142 32953178 PMC7481952

[65] Onah HE Akani CI. Rates and predictors of episiotomy in Nigerian women. Trop J Obstet Gynaecol. (2004) 21:44–5. 10.4314/tjog.v21i1.14463

[66] Woretaw E Teshome M Alene M. Episiotomy practice and associated factors among mothers who gave birth at public health facilities in Metema district, northwest Ethiopia. Reprod Health. (2021) 18:142. 10.1186/s12978-021-01194-9 34215256 PMC8252291

[67] Rasouli M Keramat A Khosravi A Mohabatpour Z. Prevalence and factors associated with episiotomy in Shahroud City, northeast of Iran. Int J Womens Health Reprod Sci. (2016) 4:125–9. 10.15296/ijwhr.2016.29

[68] Goueslard K Cottenet J Roussot A Clesse C Sagot P Quantin C. How did episiotomy rates change from 2007 to 2014? Population-based study in France. BMC Pregnancy Childbirth. (2018) 18:208. 10.1186/s12884-018-1747-8 29866103 PMC5987447

[69] Innocent N Philémon MM Prince I Justine Y Ntakwinja M Olivier N . Factors associated with episiotomy practice in Bukavu, Democratic Republic of the Congo. Int J Reprod Contracept Obstet Gynecol. (2018) 7:2553. 10.18203/2320-1770.ijrcog20182860

[70] Zilberman A Sheiner E Barrett O Hamou B Silberstein T. Once episiotomy, always episiotomy? Arch Gynecol Obstet. (2018) 298:121–4. 10.1007/s00404-018-4783-8 29785549 PMC5995988

[71] Banta D Thacker SB. The risks and benefits of episiotomy: a review. Birth. (1982) 9:25–30. 10.1111/j.1523-536X.1982.tb01599.x 6751345

[72] Steegers EA Von Dadelszen P Duvekot JJ Pijnenborg R. Pre-eclampsia. Lancet. (2010) 376:631–44. 10.1016/S0140-6736(10)60279-6 20598363

[73] English FA Kenny LC McCarthy FP. Risk factors and effective management of preeclampsia. Integr Blood Press Control. (2015) 8:7. 10.2147/IBPC.S50641 25767405 PMC4354613

[74] Malvasi A Trojano G Tinelli A Marinelli E Zaami S. Episiotomy: an informed consent proposal. J Matern Fetal Neonatal Med. (2021) 34:948–951. 10.1080/14767058.2019.1622677 31167581

[75] WHO. World Health Organisation Recommendation on Episiotomy Policy. Geneva: WHO (2018).

[76] EQUATOR Network. PRISMA 2009 checklist for reporting systematic reviews and meta-analyses. J ASEAN Fed Endocr Soc. (2015) 30:196–6.

[77] Cheng YW Caughey AB. Defining and managing normal and abnormal second stage of labor. Obstet Gynecol Clin North Am. (2017) 44:547–66. 10.1016/j.ogc.2017.08.009 29078938

[78] Gary Cunningham KJLF Bloom SL Spong CY Dashe JS Hoffman BL Casey BM . Williams Obstetrics. 26th edition. New York: Mc Graw Hill Education.

[79] Buccheri RK Sharifi C. Critical appraisal tools and reporting guidelines for evidence-based practice. Worldviews Evid.-Based Nurs. 14:463–72. 10.1111/wvn.12258 28898556

[80] Aromataris E Munn Z editors. JBI Manual for Evidence Synthesis. (2020). 10.46658/JBIMES-20-01. Available online at: https://synthesismanual.jbi.global

[81] Sim J Wright CC. The kappa statistic in reliability studies: use, interpretation, and sample size requirements. Phys Ther. (2005) 85:257–68. 10.1093/ptj/85.3.257 15733050

[82] Izuka E Dim C Chigbu C Obiora-Izuka C. Prevalence and predictors of episiotomy among women at first birth in Enugu, south east Nigeria. Ann Med Health Sci Res. (2014) 4:928–32. 10.4103/2141-9248.144916 25506488 PMC4250993

[83] Bergh JE Sueters M Segaar M Roosmalen JV. Determinants of episiotomy in rural Zimbabwe. Acta Obstet Gynecol Scand. (2003) 82:966–8. 10.1080/j.1600-0412.2003.00323.x 12956849

[84] Morhe ES Sengretsi S Danso KA. Episiotomy in Ghana. Int J Gynaecol Obstet. (2004) 86:46–7. 10.1016/j.ijgo.2004.04.006 15207675

[85] Adama O Natacha LB Smaila O Alexis SY Francoise MT Charlemagne OM . Episiotomy: epidemiological aspects, indications and prognosis in the bogodogo health district. Open J Obstet Gynecol. (2018) 08:1354–63. 10.4236/ojog.2018.813137

[86] Lai CY Cheung HW Hsi Lao TT Lau TK Leung TY. Is the policy of restrictive episiotomy generalisable? A prospective observational study. J Matern Fetal Neonatal Med. (2009) 22:1116–21. 10.3109/14767050902994820 19916709

[87] Jug Došler A Mivšek AP Verdenik I Škodič Zakšek T Levec T Petročnik P. Incidence of episiotomy in Slovenia: the story behind the numbers. Nurs Health Sci. (2017) 19:351–7. 10.1111/nhs.12352 28631876

[88] Clesse C Cottenet J Lighezzolo-Alnot J Goueslard K Scheffler M Sagot P . Episiotomy practices in France: epidemiology and risk factors in non-operative vaginal deliveries. Sci Rep. (2020) 10:20208. 10.1038/s41598-020-70881-7 33214621 PMC7677317

[89] Frankman EA Wang L Bunker CH Lowder JL. Episiotomy in the United States: has anything changed? Am J Obstet Gynecol. (2009) 200:573.e1–7. 10.1016/j.ajog.2008.11.022 19243733

[90] Singh S Thakur T Chandhiok N Dhillon BS. Pattern of episiotomy use & its immediate complications among vaginal deliveries in 18 tertiary care hospitals in India. Indian J Med Res. (2016) 143:474–80. 10.4103/0971-5916.184304 27377504 PMC4928554

[91] Kartal B Kizilirmak A Calpbinici P Demir G. Retrospective analysis of episiotomy prevalence. J Turk German Gynecol Assoc. (2017) 18:190. 10.4274/jtgga.2016.0238 29278232 PMC5776158

[92] Braga GC Clementino STP Luz PFN Scavuzzi A Noronha Neto C Amorim MMR. Fatores de risco para a episiotomia: um estudo de caso-controle. Rev Assoc Méd Bras. (2014) 60:465–72. 10.1590/1806-9282.60.05.015

[93] Trinh AT Khambalia A Ampt A Morris JM Roberts CL. Providing evidence to support obstetric practice change in Vietnam: episiotomy use among Vietnamese-born women living in Australia. Bull. World Health Organ. (2013) 91:350–356. 23678198 10.2471/BLT.12.114314 PMC3646354

[94] Ogunyemi D Manigat B Marquis J Bazargan M. Demographic variations and clinical associations of episiotomy and severe perineal lacerations in vaginal delivery. J Natl Med Assoc. (2006) 98:1874–81. 17128701 PMC2569796

[95] Belihu FB Small R Davey MA. Episiotomy and severe perineal trauma among eastern African immigrant women giving birth in public maternity care: a population based study in Victoria, Australia. Women Birth. (2017) 30:282–90. 10.1016/j.wombi.2016.11.008 27889259

[96] Hsieh W-C Liang C-C Wu D Chang S-D Chueh H-Y Chao A-S. Prevalence and contributing factors of severe perineal damage following episiotomy-assisted vaginal delivery. Taiwan J Obstet Gynecol. (2014) 53:481–5. 10.1016/j.tjog.2013.07.002 25510687

[97] Abolfazl M Hamidreza TS Narges M Maryam Y. Gestational diabetes and its association with unpleasant outcomes of pregnancy. Pak J Med Sci. (2008) 24:566–70. Available online at: https://pjms.com.pk/issues/julsep08/abstract/article

[98] Bjørstad AR Irgens-Hansen K Daltveit AK Irgens LM. Macrosomia: mode of delivery and pregnancy outcome. Acta Obstet Gynecol Scand. (2010) 89:664–9. 10.3109/00016341003686099 20235897

[99] Vitner D Bleicher I Kadour-Peero E Lipworth H Sagi S Gonen R. Does prenatal identification of fetal macrosomia change management and outcome? Arch Gynecol Obstet. (2019) 299:635–44. 10.1007/s00404-018-5003-2 30564929

[100] Manzanares S Cobo D Moreno-Martínez MD Sánchez-Gila M Pineda A. Risk of episiotomy and perineal lacerations recurring after first delivery. Birth. (2013) 40:307–11. 10.1111/birt.12077 24344712

