# Supplementary material for: Episiotomy Practice and Its Associated Factors in Africa: A Systematic Review and Meta-Analysis
Source: Front Med (Lausanne). 2022 Jun 24;9:905174. doi: 10.3389/fmed.2022.905174 (PMC9295659; doi:10.3389/fmed.2022.905174)
Supplement: Supplementary file 4 [file Table_4.DOCX]

**Search details for Episiotomy practice and its associated factors in Africa, 2021**

| Concept map1 | Women who give birth | 1.Key word | “Women*” [tw] OR “pregnant mother” [tw] OR “birth” [tw] |
| --- | --- | --- | --- |
|  |  | 2.Mesh term | "Pregnant Women"[Mesh] AND "Women"[Mesh] |
| Concept map2 | Episiotomy practice | 3.Key word | “Episiotomy ”[tw] OR “ Episiotomy practice ”[tw] |
|  |  | 4.Mesh term | ("Obstetric Surgical Procedures"[Mesh]) AND "Episiotomy"[Mesh] |
| Concept map3 | Associated factors | 5.Key word | “Associated factors”[tw] OR “ risk factors”[tw] |
|  |  | 6.Mesh term | "Risk Factors"[Mesh] |
| Combined | ("women*"[Text Word] OR "pregnant mother"[Text Word] OR "birth"[Text Word] OR "mobile technology"[Text Word] OR ("Pregnant Women"[MeSH Terms] AND "Women"[MeSH Terms])) AND ("Episiotomy"[Text Word] OR "Episiotomy practice"[Text Word] OR ("Obstetric Surgical Procedures"[MeSH Terms] AND "Episiotomy"[MeSH Terms])) AND ("Associated factors"[Text Word] OR "Risk Factors"[Text Word] OR "Risk Factors"[MeSH Terms]) | | |
