# Supplementary material for: Episiotomy Practice and Its Associated Factors in Africa: A Systematic Review and Meta-Analysis
Source: Front Med (Lausanne). 2022 Jun 24;9:905174. doi: 10.3389/fmed.2022.905174 (PMC9295659; doi:10.3389/fmed.2022.905174)
Supplement: Supplementary file 5 [file Table_5.DOCX]

Quality assessment of studies using JBI’s critical appraisal tools designed for descriptive cross-sectional study

| Study | Sample size | JBI’s critical appraisal questions | | | | | | | | | Score | Overall Appraisal |
| --- | --- | --- | --- | --- | --- | --- | --- | --- | --- | --- | --- | --- |
|  |  | Q1 | Q2 | Q3 | Q4 | Q5 | Q6 | Q7 | Q8 | Q9 |  |  |
| Bergh et al. | 3589 | Y | Y | Y | Y | y | Y | Y | Y | Y | 9 | Included |
| Morhe et al. | 2151 | Y | Y | Y | Y | Y | Y | Y | U | Y | 9 | Included |
| Adama et al. | 3703 | Y | Y | Y | Y | Y | Y | Y | Y | Y | 9 | Included |

Y –Yes; N- No; U –Unclear;Q- Question. Overall score is calculated by counting the number of Y’s in each row.Q1=Was the sample frame appropriate to address the target population? Q2=Were study participants sampled in an appropriate way? Q3=Was the sample size adequate? Q4=Were the study subjects and the setting described in detail? Q5=Was the data analysis conducted with sufficient coverage of the identified sample? Q6=Were valid methods used for the identification of the condition? Q7=Was the condition measured in a standard, reliable way for all participants? Q8=Was there appropriate statistical analysis? Q9=Was the response rate adequate, and if not, was the low response rate managed appropriately?
