# Supplementary material for: Episiotomy Practice and Its Associated Factors in Africa: A Systematic Review and Meta-Analysis
Source: Front Med (Lausanne). 2022 Jun 24;9:905174. doi: 10.3389/fmed.2022.905174 (PMC9295659; doi:10.3389/fmed.2022.905174)
Supplement: Supplementary file 6 [file Table_6.DOCX]

Quality assessment of studies using JBI’s critical appraisal tools designed for analytical cross-sectional study.

| Study | Sample size | JBI’s critical appraisal questions | | | | | | | | Score | Overall Appraisal |
| --- | --- | --- | --- | --- | --- | --- | --- | --- | --- | --- | --- |
|  |  | Q1 | Q2 | Q3 | Q4 | Q5 | Q6 | Q7 | Q8 |  |  |
| Yemaneh et al. | 338 | Y | y | y | y | y | y | y | y | 8 | Included |
| Woretaw et al. | 410 | y | y | y | y | y | y | y | y | 8 | Included |
| Kidane et al. | 407 | y | y | y | y | y | y | y | y | 8 | Included |
| Solomon et al. | 387 | Y | y | y | y | y | y | y | y | 8 | Included |
| Beyene et al. | 411 | y | y | y | y | y | y | y | y | 8 | Included |
| Fikadu et al. | 400 | Y | y | y | y | y | y | y | y | 8 | Included |
| Tobiaw et al. | 405 | y | y | y | y | y | y | y | y | 8 | Included |
| Teshome et al. | 306 | y | y | y | y | N | y | y | y | 7 | Included |
| Okeke et al. | 3032 | y | y | y | y | y | y | y | y | 8 | Included |
| Alayande et al. | 280 | Y | U | y | y | y | y | y | y | 7 | Included |
| Onah et al. | 433 | y | y | y | y | y | y | y | y | 8 | Included |
| Izuka et al. | 662 | Y | y | y | y | y | y | y | y | 8 | Included |
| Chigbu et al. | 4174 | y | y | y | y | y | y | y | y | 8 | Included |
| Owa et al. | 728 | Y | y | y | y | y | y | y | y | 8 | Included |
| Ayyuba et al. | 12,168 | y | y | y | y | y | y | y | Y | 8 | Included |
| Enyindah etal. | 4720 | Y | y | y | y | y | y | y | y | 8 | Included |
| Pebolo et al. | 249 | y | U | y | y | y | y | y | y | 7 | Included |
| Innocent et al . | 1878 | y | y | y | y | y | y | y | y | 8 | Included |

Y –Yes; N- No; U –Unclear;Q- Question. Overall score is calculated by counting the number of Y’s in

For analytical cross-sectional study, the JBI checklist assessed the following questions Q1= were the criteria for inclusion in the sample clearly defined? Q2= were the study subjects and the= Were objective, standard criteria used for measurement of the condition? Q5= Were confounding factors identified? Q6= Were strategies to deal with confounding factors stated? Q7= Were the outcomes measured in a valid and reliable way? Q8= Was appropriate statistical analysis used?
